# Sarcopenia modifies the associations of nonalcoholic fatty liver disease with all-cause and cardiovascular mortality among older adults

**DOI:** 10.1038/s41598-021-95108-1

**Published:** 2021-08-02

**Authors:** Xingxing Sun, Zhelong Liu, Fuqiong Chen, Tingting Du

**Affiliations:** 1 Department of Anesthesiology, Tongji Hospital, Tongji Medical College, Huazhong University of Science and Technology, Wuhan, 430030 China; 2 Department of Endocrinology, Tongji Hospital, Tongji Medical College, Huazhong University of Science and Technology, Wuhan, 430030 Hubei Province China; 3 Branch of National Clinical Research Center for Metabolic Diseases, Wuhan, Hubei China

**Keywords:** Disease prevention, Public health

## Abstract

The contribution of nonalcoholic fatty liver disease (NAFLD) to all-cause and cardiovascular mortality remains controversial. Sarcopenia, a measure of muscle mass, strength and function, may identify which persons are most at risk for adverse effects of NAFLD. We aimed to test the hypothesis that sarcopenia modifies the associations between NAFLD and all-cause and cardiovascular mortality. A total of 2446 older adults (≥ 60 years) from the third National Health and Nutrition Examination Survey were enrolled. Their mortality data were linked to death certificates in the National Death Index. Sarcopenia was defined as having low skeletal muscle mass together with slow gait speed, which captures both muscle mass and muscle function. Ultrasound tests were used for the assessment of hepatic steatosis. During follow-up (median 16.8 years), 1530 older subjects died from any cause, of which 379 were cardiovascular-related. All-cause and cardiovascular mortality rates were 4.31 and 1.07 per 100 person-years, respectively. In a multivariate model, using participants without NAFLD and sarcopenia as the reference group, individuals with both NAFLD and sarcopenia had 1.69 times [95% confidence interval (CI) 1.23–2.31] and 2.17 times (95% CI 1.33–3.54) higher risks of all-cause and cardiovascular mortality, respectively. However, NAFLD persons without sarcopenia had hazard ratios for all-cause and cardiovascular mortality similar to those of the reference group. Sarcopenia modified the associations of NAFLD with all-cause and cardiovascular mortality. Sarcopenia may identify older adults who are at the highest risk for adverse outcomes associated with NAFLD.

## Introduction

Nonalcoholic fatty liver disease (NAFLD) is associated with metabolic disorders such as hyperglycemia and dyslipidemia^1,2^. There has been a large body of epidemiological evidence showing the relationship of NAFLD with all-cause and cardiovascular mortality, but the results are contradictory^3–5^. This may be caused by the existing substantial heterogeneity in health status among patients with NAFLD. High quantities of skeletal muscle mass, an aspect of health status, have a strong protective effect against mortality^6,7^. Therefore, high quantities of muscle mass may attenuate or eliminate the increased mortality risk associated with NAFLD. NAFLD and sarcopenia, which is characterized by a progressive loss of skeletal muscle mass, strength, and function, share the insulin resistance background^8,9^. Moreover, sarcopenia may be involved in the pathogenesis of NAFLD by reducing energy expenditure. Increased secretion of proinflammatory cytokines in the state of NAFLD may reduce muscle protein synthesis and promote muscle protein breakdown^10,11^, and thus induce sarcopenia. A clear understanding of the combined effect of NAFLD and sarcopenia on all-cause and cardiovascular mortality risk is needed to aid in risk stratification and in turn target patients at the highest risk of all-cause and cardiovascular mortality. Hence, we aimed to investigate the combined relationship of NAFLD and sarcopenia with all-cause and cardiovascular mortality to understand whether sarcopenia modified the association of NAFLD with all-cause and cardiovascular mortality.

## Materials and methods

### Study population

The National Health and Nutrition Examination Surveys (NHANES) are a series of cross-sectional health examination surveys conducted by the National Center for Health Statistics (NCHS) of the Centers for Disease Control and Prevention. All mortality data from each NHANES were ascertained by the NCHS from National Death Index (NDI) death certificate records. We chose NHANES III as the baseline since we had detailed information regarding the assessment of hepatic steatosis. Full details of the survey have been described elsewhere^12^. Briefly, the survey followed a complex stratified, multistage probability cluster sampling design to ensure that the sample is nationally representative of the civilian, noninstitutionalized US population. Participants were interviewed at home for basic sociodemographic and health-related information. After the in-home interview, participants are invited to attend a mobile examination center, where they underwent a set of standardized physical examinations and laboratory measurements. The survey procedures were reviewed and approved by the National Center for Health Statistics ethics review board in accordance with the ethical standards laid down in the 1964 Declaration of Helsinki and its later amendments. Informed consent was obtained from all participants and/or their legal guardians.

**Figure 1:**
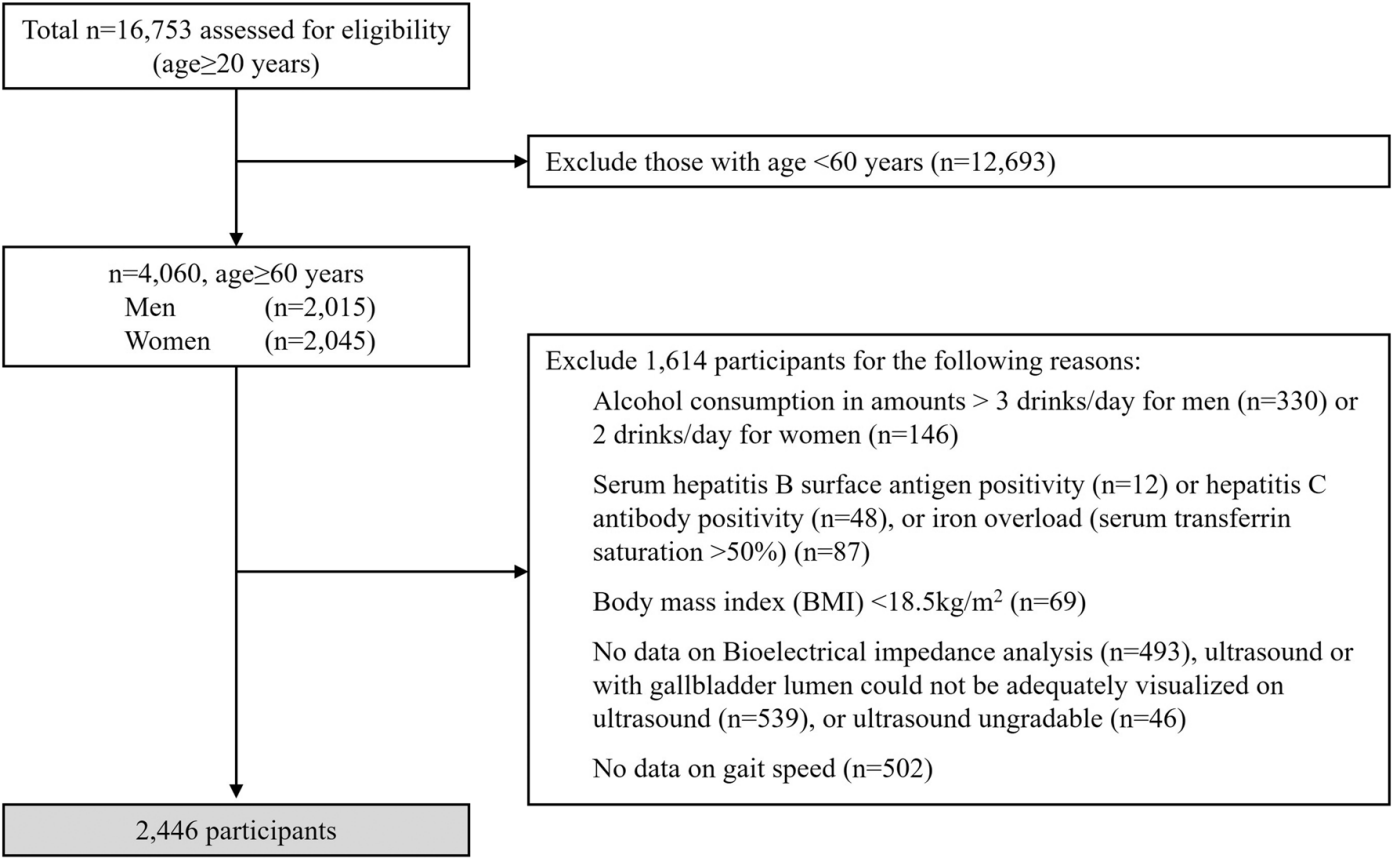
Flow diagram of subject inclusion and exclusion in the NHANES III.

We restricted our analyses to individuals aged 60 years and older who completed the gallbladder ultrasound examination and had bioelectrical impedance data and gait speed measures (Fig. 1). Our rationale for restricting our sample to individuals aged ≥ 60 years was that the prevalence of sarcopenia is more prevalent in this population^13^. We excluded 476 participants with alcohol consumption in amounts > 3 drinks/day for men (330) or > 2 drinks/day for women (146), 12 participants with serum hepatitis B surface antigen positivity, 48 participants with hepatitis C antibody positivity, 87 participants with iron overload (serum transferrin saturation > 50%), 69 participants with body mass index (BMI) < 18.5 kg/m^2^, 493 participants without data on Bioelectrical impedance analysis, 502 participants without data on gait speed, 539 participants with gallbladder lumen could not be adequately visualized on ultrasound, and 46 participants with ultrasound ungradable. The remaining available 2446 participants were included in our data analysis.

### Anthropometric and biochemical measurements

As elaborated in our previous report^14^, BMI was calculated as weight (in kilograms) divided by the square of height (in meters). Waist circumference (WC) was measured with a steel measuring tape just above the iliac crest to the nearest 1 mm. Three or four blood pressure (BP) measurements were taken in sequence on seated participants using mercury sphygmomanometers. The last two readings were averaged.

Methods used for laboratory evaluations have been described in detail elsewhere^14^. Homeostasis model assessment of insulin resistance (HOMA-IR) was calculated by the formula: HOMA-IR = fasting insulin (micro-international units per milliliter) × fasting glucose (millimoles per liter)/22.5.

### Assessment of NAFLD

Ultrasound tests (Toshiba SSA-90A, Tustin, CA) were used for the assessment of hepatic steatosis. Archived videotapes on gallbladder ultrasounds were reviewed between 2009 and 2010 to ascertain the presence of fat within the hepatic parenchyma. The diagnosis of fatty liver was based on the following five criteria^15^: the brightness of liver parenchyma, presence of liver to kidney contrast, presence of echogenic walls within the small intrahepatic vessels, presence of deep beam attenuation, and definition of gallbladder walls, which were also described in our previous report^14^. NAFLD was initially categorized as a 4-level classification (none, mild, moderate, or severe) and then recorded as a 2-level classification (none to mild or moderate to severe), which was the classification used for the current analysis. For the two-level hepatic steatosis categorization, the intrarater and interrater k statistics were 0.77 [95% confidence interval (CI) 0.73–0.82] and 0.70 (0.64–0.76), respectively.

Regarding fibrosis in NAFLD, Fibrosis‐4 (FIB‐4) score was calculated by the following formula: FIB-4 = (age [years] * aspartate aminotransferase [U/L])/(platelet count [10^9^/L] *(alanine transaminase [U/L])^1/2^)^16^. The higher the value, the more likely to suffer from advances fibrosis.

### Definition of sarcopenia

Bioelectrical impedance analysis (BIA) was assessed using a Valhalla 1990B Bio-Resistance Body Composition Analyzer (Valhalla Scientific, San Diego, CA, USA). All subjects fasted for a minimum of 6 h. A single tetrapolar measurement of resistance was taken between the right wrist and ankle while lying in the supine position. Skeletal muscle mass was calculated using a validated formula^17^: Skeletal muscle mass (kg) = [(height^2^/BIA-resistance × 0.401) + (gender × 3.825) + (age × −0.071)] + 5.102, where height is expressed in cm, BIA-resistance is expressed in ohms, gender is equal to 1 for men and 0 for women, and age is expressed in years. The skeletal muscle index (SMI, kg/m^2^) is the skeletal muscle mass (kg) indexed for height^2^ (in meters). Low muscle mass is defined as SMI ≤ 10.75 kg/m^2^ for men and ≤ 6.74 kg/m^2^ for women^18^. These SMI thresholds are based on disability risk and are recommended to identify sarcopenia^18^.

It has demonstrated that slow gait speed reflect functional capacity and subclinical health impairment in the older adults^19–21^. A timed 8-foot walk test was performed twice in NHANES III. The participant was asked to walk at their usual pace. The faster of the two trials were used in this analysis. Timing began when the participant’s first foot stepped over the starting line and ended when one of the feet crossed over the finish line. The 8-foot speed was converted to a 4-m equivalent by adopting an established formula^22^. Slow gait speed is defined as a gait speed ≤ 0.8 m/s^18^. This cutoff is associated with adverse health outcomes^23^.

Muscle strength measurements were not available in the NHANES III. Based on the retrospective longitudinal study design, we define sarcopenia in the older adults as slow gait speed, a measure of muscle function, together with low muscle mass^18^.

### Definition of sarcopenia obesity

Evidence suggests that fat mass to fat-free mass ratio (FM/FFM) can provide a robust measure of body composition changes^24^. Since specific cut-off points for diagnosing obesity according to FM/FFM are not completely uniform, increased FM/FFM is defined as FM/FFM ≥ 3/4 percentile in the present study. Sarcopenia obesity was defined as low muscle mass, increased FM/FFM, as well as slow gait speed.

### Mortality data

Mortality data including causes of death were available from the date of NHANES III survey participation (1988–1994) through December 31, 2011, using a probabilistic match that linked NHANES III participants with NDI death certificate records. The NCHS indicated that 96.1% of deceased individuals and 99.4% of living individuals were correctly classified using this matching methodology^25^. Causes of death for those dying prior to 1998 were determined according to the 9th revision of the International Statistical Classification of Disease, Injuries, and Causes of Death (ICD-9) guidelines. After 1998, they were determined by the 10th revision (ICD-10) guidelines^26^. The present study focused on all-cause mortality and cardiovascular mortality (ICD-10 I00-09, I11, I13, I20-51, and I60-69).

### Statistical analysis

Complex survey procedures in SAS 9.2 (SAS Institute, Inc., Cary, NC) were performed for all analyses. Sample weights were incorporated to produce nationally representative estimates. Participants were divided into 4 mutually exclusive groups based on the cross-classification of NAFLD (with and without NAFLD) and sarcopenia status (with and without sarcopenia). Continuous variables were presented as means ± standard errors (SE). Logarithmic transformation was performed where needed. Categorical variables were presented as percentages. ANOVA was applied to compare differences in means between groups. A Chi-square test was performed to assess differences in proportions between groups. We divided FIB-4 into quartiles. SMI values were evaluated in quartiles of FBI-4. Correlations between SMI values and FIB-4 scores were calculated using Spearman correlation. Follow-up time was from the date of the NHANES III examination to the date of death or December 31, 2011, whichever came first. Hazard ratios (HRs) and corresponding 95% confidence intervals (CI) were estimated with the use of cox regression analysis. Proportional hazard assumption was adjusted for all potential mortality predictors. Cox proportional hazards models were used to examine the joint associations of NAFLD and sarcopenia with mortality risk to determine whether the associations of NAFLD with mortality risk was modified by sarcopenia status. The six models were as follows: Model 1 was adjusted for age, gender, race-ethnicity, and education level. Model 2 was adjusted for smoking and drinking status, body mass index, hypertension, and diabetes in addition to the factors included in model 1. Model 3 was adjusted for all the variables in model 2 plus total cholesterol, triglyceride, and HDL-cholesterol. Model 4 was adjusted for all the variables in model 3 plus C-reactive protein. Model 5 was adjusted for HOMA-IR in addition to the factors included in model 3. Model 6 was adjusted for age, gender, race-ethnicity, education level, smoking and drinking status, body mass index, total cholesterol, triglyceride, HDL-cholesterol, nutritional status^27^, number of medications, and number of comorbidities, including stroke, asthma, chronic obstructive pulmonary disease thyroid disease, lupus, gout, cancer, hypertension, hyperlipidemia, fracture, osteoporosis, gallbladder disease, urolithiasis, diabetes, and coronary heart disease. For analysis of sarcopenia-related risks of all-cause and cardiovascular mortality, model 7 was adjusted for all the variables in model 6 plus NAFLD. For analysis of NAFLD-related risks of all-cause and cardiovascular mortality, model 7 was adjusted for all the variables in model 6 plus sarcopenia. We chose these variables because of their potential role as confounders from a clinical point-of-view. Significance was accepted at a two-tailed *P* < 0.05.

## Results

Among individuals aged equal or above 60 years old, 2446 participants taken the 8-foot walk test, 502 did not taken the test. Participants were more often female than nonparticipants (42.4 vs. 51.7, *P* = 0.032). Similar age (66.6 ± 0.1 vs. 66.2 ± 0.4, *P* = 0.199), race/ethnicity, most of the studied risk factors, and SMI (8.4 ± 0.1 vs. 8.8 ± 0.2, *P* = 0.23) were observed between subjects who had and did not have gait speed data (supplementary Table S1).

During follow-up (median 16.8 years), 1530 (62.6%) older adults died from any cause, of which 379 (15.5%) were cardiovascular-related. All-cause and cardiovascular mortality rates were 4.31 and 1.07 per 100 person-years, respectively.

BMI, WC, systolic BP, plasma glucose, HbA1c, triglycerides, and HOMA-IR were significantly higher, whereas high density lipoprotein cholesterol (HDL-C) was lower in NAFLD individuals than counterparts without NAFLD (Table 1). Compared with individuals without sarcopenia, sarcopenic counterparts were more likely to be older, less educated. HOMA-IR and C-Reactive Protein (CRP) were significantly higher, whereas BMI and gait speed were lower in sarcopenic individuals than counterparts without sarcopenia (Table 1).

**Table 1:** Characteristics of the study participants according to the presence of nonalcoholic fatty liver disease or sarcopenia. Data are presented as means ± standard errors or percent.

	Without NAFLD	With NAFLD	*P* (NAFLD vs without NAFLD)	Without sarcopenia	With sarcopenia	*P* (Sarcopenia vs without Sarcopenia
N	1732	714		2022	444	
Age, years	66.6 ± 0.1	66.6 ± 0.2	0.711	66.4 ± 0.2	67.9 ± 0.4	0.001
Men, %	41.4	45.0	0.175	40.8	56.6	< 0.001
**Race-ethnicity, %**						
Non-Hispanic white	82.9	86. 0	0.839	84.4	80.0	0.143
Non-Hispanic black	8.8	6.2	0.004	7.5	11.4	0.053
Mexican American	2.3	3.4	0.028	2.5	3.4	0.071
Smoking, %	58.5	60.8	0.518	56.7	74.4	< 0.001
Education, years	11.6 ± 0.2	11.1 ± 0.2	0.002	11.6 ± 0.2	10.4 ± 0.3	< 0.001
Body mass index, kg/m^2^	26.5 ± 0.2	30.0 ± 0.3	< 0.001	27.8 ± 0.2	25.6 ± 0.3	< 0.001
Waist circumference, cm	95.2 ± 0.5	104.8 ± 0.6	< 0.001	98.2 ± 0.4	96.5 ± 0.9	0.106
Systolic blood pressure, mmHg	134.5 ± 0.6	140.0 ± 0.9	< 0.001	135.8 ± 0.6	137.6 ± 1.5	0.322
Diastolic blood pressure, mmHg	74.8 ± 0.3	75.3 ± 0.5	0.299	74.9 ± 0.3	74.8 ± 0.8	0.860
Plasma glucose, mmol/l	5.6 ± 0.1	6.5 ± 0.1	< 0.001	5.9 ± 0.1	5.9 ± 0.1	0.703
HbA1c, %	5.7 ± 0.0	6.2 ± 0.1	< 0.001	5.8 ± 0.0	5.8 ± 0.1	0.643
Total cholesterol, mmol/l	5.8 ± 0.0	5.9 ± 0.1	0.181	5.9 ± 0.0	5.8 ± 0.1	0.706
Triglycerides, mmol/l	1.7 ± 0.0	2.5 ± 0.1	< 0.001	2.0 ± 0.0	1.9 ± 0.1	0.393
HDL-cholesterol, mmol/l	1.4 ± 0.0	1.2 ± 0.0	< 0.001	1.3 ± 0.0	1.4 ± 0.0	0.173
LDL-cholesterol, mmol/l	3.7 ± 0.1	3.6 ± 0.1	0.218	3.7 ± 0.0	3.6 ± 0.1	0.430
C-reactive protein, mg/l	1.0 ± 0.1	1.0 ± 0.1	0.932	1.0 ± 0.1	1.3 ± 0.1	0.037
HOMA-IR	3.0 ± 0.2	6.0 ± 0.5	< 0.001	3.9 ± 0.2	3.7 ± 0.3	0.451
Gait speed, m/s	0.9 ± 0.0	0.9 ± 0.0	0.344	0.9 ± 0.0	0.7 ± 0.0	< 0.001
Skeletal muscle index, kg/m^2^	8.8 ± 0.1	8.2 ± 0.1	< 0.001	8.5 ± 0.1	8.0 ± 0.1	0.003

*NAFLD* nonalcoholic fatty liver disease, *HbA1c* hemoglobin A1c, *HDL* high density lipoprotein, *LDL* low density lipoprotein, *HOMA-IR* homeostasis model assessment of insulin resistance.

The effects of NAFLD and sarcopenia on all-cause and cardiovascular mortality in multivariate-adjusted models were presented in Table 2, respectively. NAFLD patients had hazard ratios (HR) for all-cause and cardiovascular mortality similar to those without NAFLD. Sarcopenia carried a greater risk of all-cause (HR 1.47, 95% CI 1.20–1.80) and cardiovascular (HR 1.73, 95% CI 1.20–2.48) mortality. Adjustment for NAFLD, chronic conditions, CRP, and HOMA-IR did not markedly change these associations.

**Table 2:** Hazard ratios (with 95% confidence intervals) for sarcopenia- or nonalcoholic fatty liver disease (NAFLD)-related risks of all-cause and cardiovascular mortality.

	Sarcopenia		With NAFLD	
**All-cause mortality**				
Model 1	1.47 (1.20–1.80)	*P* < 0.001	1.0 (0.85–1.17)	*P* = 0.979
Model 2	1.46 (1.20–1.78)	*P* < 0.001	0.85 (0.72–1.10)	*P* = 0.052
Model 3	1.49 (1.23–1.81)	*P* < 0.001	0.85 (0.71–1.20)	*P* = 0.058
Model 4	1.47 (1.22–1.79)	*P* < 0.001	0.85 (0.72–1.01)	*P* = 0.071
Model 5	1.51 (1.25–1.83)	*P* < 0.001	0.83 (0.70–0.98)	*P* = 0.030
Model 6	1.45 (1.19–1.70)	*P* < 0.001	0.89 (0.75–1.05)	*P* = 0.196
Model 7	1.44 (1.18–1.75)	*P* < 0.001	0.90 (0.76–1.06)	*P* = 0.245
**Cardiovascular mortality**				
Model 1	1.73 (1.20–2.48)	*P* = 0.003	1.16 (0.86–1.57)	*P* = 0.314
Model 2	1.72 (1.19–2.49)	*P* = 0.004	0.98 (0.72–1.33)	*P* = 0.923
Model 3	1.87 (1.29–2.72)	*P* = 0.001	0.91 (0.67–1.24)	*P* = 0.586
Model 4	1.81 (1.24–2.63)	*P* = 0.027	0.91 (0.67–1.24)	*P* = 0.572
Model 5	1.92 (1.32–2.78)	*P* < 0.001	0.88 (0.65–1.21)	*P* = 0.457
Model 6	1.78 (1.24–2.55)	*P* = 0.002	1.00 (0.73–1.37)	*P* = 0.969
Model 7	1.78 (1.24–2.56)	*P* = 0.002	1.02 (0.75–1.39)	*P* = 0.875

To assess the hazard ratios (with 95% confidence intervals) for sarcopenia-related risks of all-cause and cardiovascular mortality, subjects without sarcopenia were served as the reference group. To assess the hazard ratios (with 95% confidence intervals) for NAFLD-related risks of all-cause and cardiovascular mortality, subjects without NAFLD were served as the reference group.

Model 1 was adjusted for age, gender, race-ethnicity, and education level.

Model 2 was adjusted for smoking and drinking status, body mass index, hypertension, and diabetes in addition to the factors included in model 1.

Model 3 was adjusted for total cholesterol, triglyceride, and HDL-cholesterol in addition to the factors included in model 2.

Model 4 was adjusted for C-reactive protein in addition to the factors included in model 3.

Model 5 was adjusted for HOMA-IR in addition to the factors included in model 3.

Model 6 was adjusted for comorbid conditions such as stoke, coronary heart disease, and chronic obstructive pulmonary disease in addition to the factors included in model 3.

For analysis of sarcopenia-related risks of all-cause and cardiovascular mortality, model 7 was adjusted for all the variables in model 6 plus NAFLD.

For analysis of NAFLD-related risks of all-cause and cardiovascular mortality, model 7 was adjusted for all the variables in model 6 plus sarcopenia.

SMI showed a strong negative relationship with FIB-4 (supplementary Figure S1). The correlation coefficient of SMI with FIB-4 was − 0.24 (*P* < 0.001).

Table 3 listed the joint associations of NAFLD and sarcopenia with all-cause and cardiovascular mortality. Compared with the reference group (those without NAFLD and sarcopenia), the sex-, age-, ethnicity-, and education-adjusted HR (95% CI) of all-cause mortality was 1.35 (1.06–1.70) for participants with sarcopenia and without NAFLD, 0.97 (0.81–1.15) for participants with NAFLD and without sarcopenia, and 1.91 (1.39–2.64) for participants with both NAFLD and sarcopenia. The association of the combined NAFLD and sarcopenia with increased all-cause mortality risk persisted after additional adjustment for BMI, smoking and drinking habits, hypertension, and diabetes status (Model 2). Further adjustment for total cholesterol (TC), triglyceride, and HDL-C did not change the association (Model 3). The addition of inflammation (CRP) did not significantly reduce the HR (Model 4). Since insulin resistance predicted increased mortality risk, we performed an additional adjustment for HOMA-IR, and a similar association was observed (Model 5). Because nutritional status, number of medications, and number of comorbidities were closely associated with all-cause mortality, a multivariate analysis adjusted for these factors showed that the significant association remained (Model 6). The combined association of NAFLD and sarcopenia with cardiovascular mortality showed similar patterns (Table 3). The interactions between NAFLD and sarcopenia on all-cause and cardiovascular mortality showed statistical significance, indicating that the associations of NAFLD with all-cause and cardiovascular mortality (all *P* values < 0.05) differed by sarcopenia status. We further analyzed the relationship between sarcopenia obesity and all-cause mortality risk (Supplementary Table S2). We found a role of sarcopenia obesity on all-cause mortality.

**Table 3:** All-cause and cardiovascular mortality during 16.8 years of follow-up according to combinations of nonalcoholic fatty liver disease (NAFLD) and sarcopenia status.

	Without NAFLD				NAFLD		Interaction
	Sarcopenia		Without sarcopenia		Sarcopenia		*P*
**All-cause mortality**							
Model 1	1.35 (1.06–1.70)	*P* = 0.012	0.97 (0.81–1.15)	*P* = 0.742	1.91 (1.39–2.64)	*P* < 0.001	0.056
Model 2	1.30 (1.03–1.64)	*P* = 0.024	0.80 (0.67–0.95)	*P* = 0.015	1.64 (1.21–2.22)	*P* = 0.002	0.025
Model 3	1.32 (1.0–1.65)	*P* = 0.014	0.80 (0.67–0.96)	*P* = 0.019	1.67 (1.21–2.30)	*P* = 0.002	0.026
Model 4	1.30 (1.05–1.65)	*P* = 0.014	0.81 (0.67–0.97)	*P* = 0.026	1.62 (1.17–2.24)	*P* = 0.003	0.02
Model 5	1.35 (1.07–1.69)	*P* = 0.009	0.78 (0.65–0.94)	*P* = 0.009	1.62 (1.17–2.24)	*P* = 0.003	0.034
Model 6	1.39 (1.07–1.81)	*P* = 0.011	0.90 (0.73–1.10)	*P* = 0.364	1.66 (1.19–2.33)	*P* = 0.021	0.041
**Cardiovascular mortality**							
Model 1	1.58 (1.01–2.48)	*P* = 0.042	1.12 (0.80–1.5)	*P* = 0.479	2.62 (1.53–4.48)	*P* < 0.001	0.029
Model 2	1.51 (0.95–2.38)	*P* = 0.078	0.91 (0.64–1.29)	*P* = 0.614	2.22 (1.33–3.70)	*P* = 0.002	0.017
Model 3	1.65 (1.05–2.61)	*P* = 0.030	0.86 (0.61–1.23)	*P* = 0.423	2.22 (1.29–3.80)	*P* = 0.004	0.022
Model 4	1.62 (1.02–2.56)	*P* = 0.040	0.87 (0.60–1.23)	*P* = 0.436	2.0 (1.20–3.58)	*P* = 0.007	0.029
Model 5	1.70 (1.08–2.70)	*P* = 0.022	0.84 (0.59–1.19)	*P* = 0.338	2.16 (1.26–3.68)	*P* = 0.005	0.026
Model 6	1.71 (1.04–2.80)	*P* = 0.034	0.99 (0.66–1.47)	*P* = 0.961	2.13 (1.16–3.94)	*P* = 0.015	0.001

Subjects without sarcopenia or NAFLD were served as the reference group.

Data were presented as hazard ratios (with 95% confidence intervals).

Model 1 was adjusted for age, gender, race-ethnicity, and education level.

Model 2 was adjusted for smoking and drinking status, body mass index, hypertension, and diabetes in addition to the factors included in model 1.

Model 3 was adjusted for total cholesterol, triglyceride, and HDL-cholesterol in addition to the factors included in model 2.

Model 4 was adjusted for C-reactive protein in addition to the factors included in model 3.

Model 5 was adjusted for HOMA-IR in addition to the factors included in model 3.

Model 6 was adjusted for age, gender, race-ethnicity, education level, smoking and drinking status, body mass index, total cholesterol, triglyceride, HDL-cholesterol, nutritional status, number of medications, and number of comorbidities.

**Figure 2:**
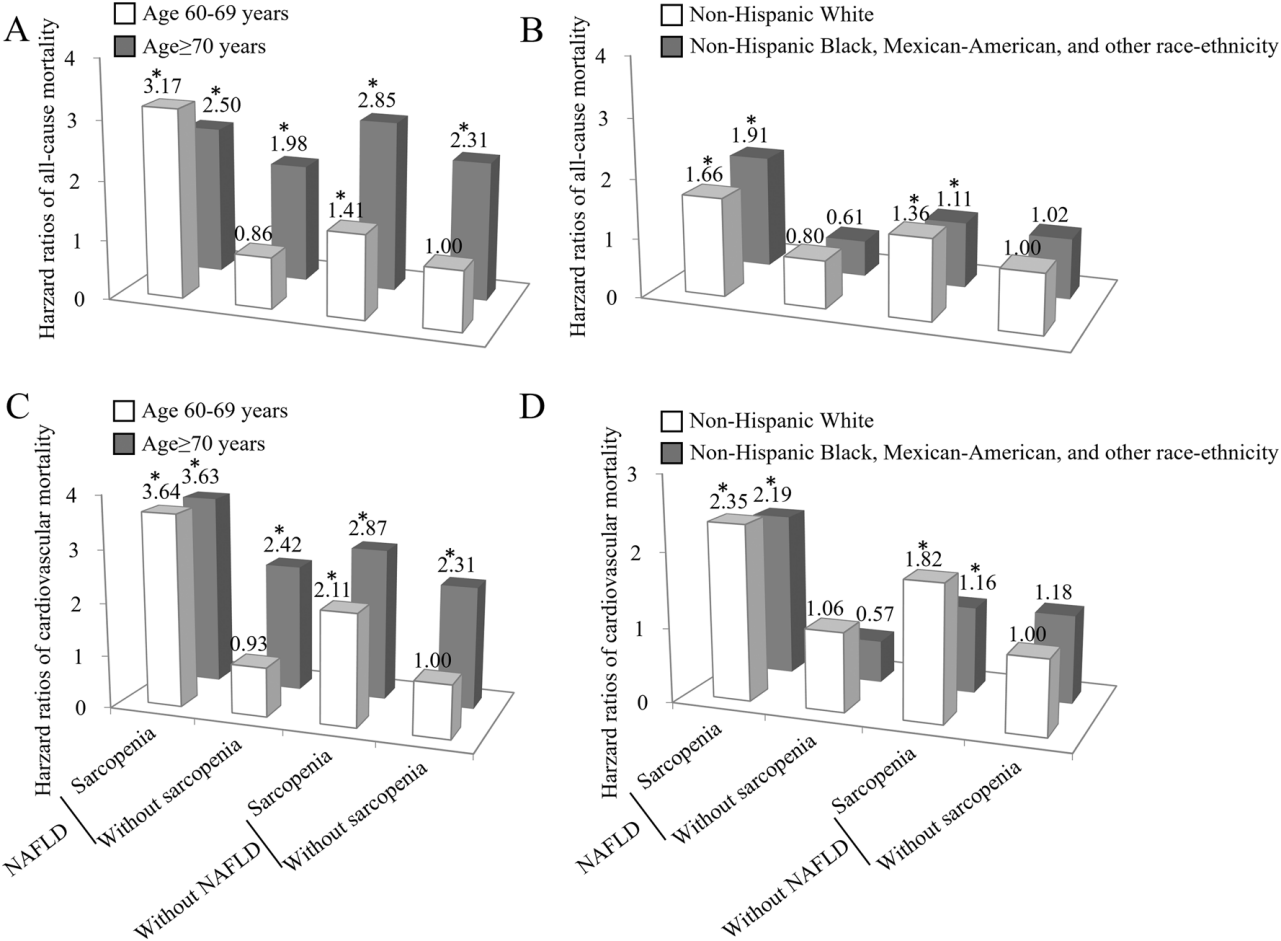
Joint effects of nonalcoholic fatty liver disease (NAFLD) and sarcopenia on all-cause mortality (upper panel) and cardiovascular mortality (lower panel) after stratification for age, and race-ethnicity. *All-cause and cardiovascular mortality risks were significantly higher in sarcopenic subjects with NAFLD regardless of the age group and race-ethnicity. For the age subgroup, hazard ratios (95% confidence intervals) were adjusted for gender, race-ethnicity, education level, smoking and drinking status, body mass index, total cholesterol, triglyceride, HDL-cholesterol, and comorbid conditions such as stoke, coronary heart disease, and chronic obstructive pulmonary disease. For the race-ethnicity subgroup, hazard ratios (95% confidence intervals) were adjusted for gender, age, education level, smoking and drinking status, body mass index, total cholesterol, triglyceride, HDL-cholesterol, and comorbid conditions such as stoke, coronary heart disease, and chronic obstructive pulmonary disease. Statistics and *P* values were showed in Supplementary Table S3.

To take into account the potential confounder of age and race-ethnicity, we stratified the study population by age group (60–69 and ≥ 70 years) and race-ethnicity. NAFLD patients with sarcopenia in both age groups had significantly higher risks of all-cause and cardiovascular mortality (Fig. 2). Further, sarcopenia was associated with an equal or higher risk of cardiovascular mortality among individuals aged ≥ 70 years (Fig. 2), indicating that sarcopenia was associated with cardiovascular mortality independent of age. NAFLD patients with sarcopenia conferred increased risks of all-cause and cardiovascular mortality, irrespective of race-ethnicity.

**Figure 3:**
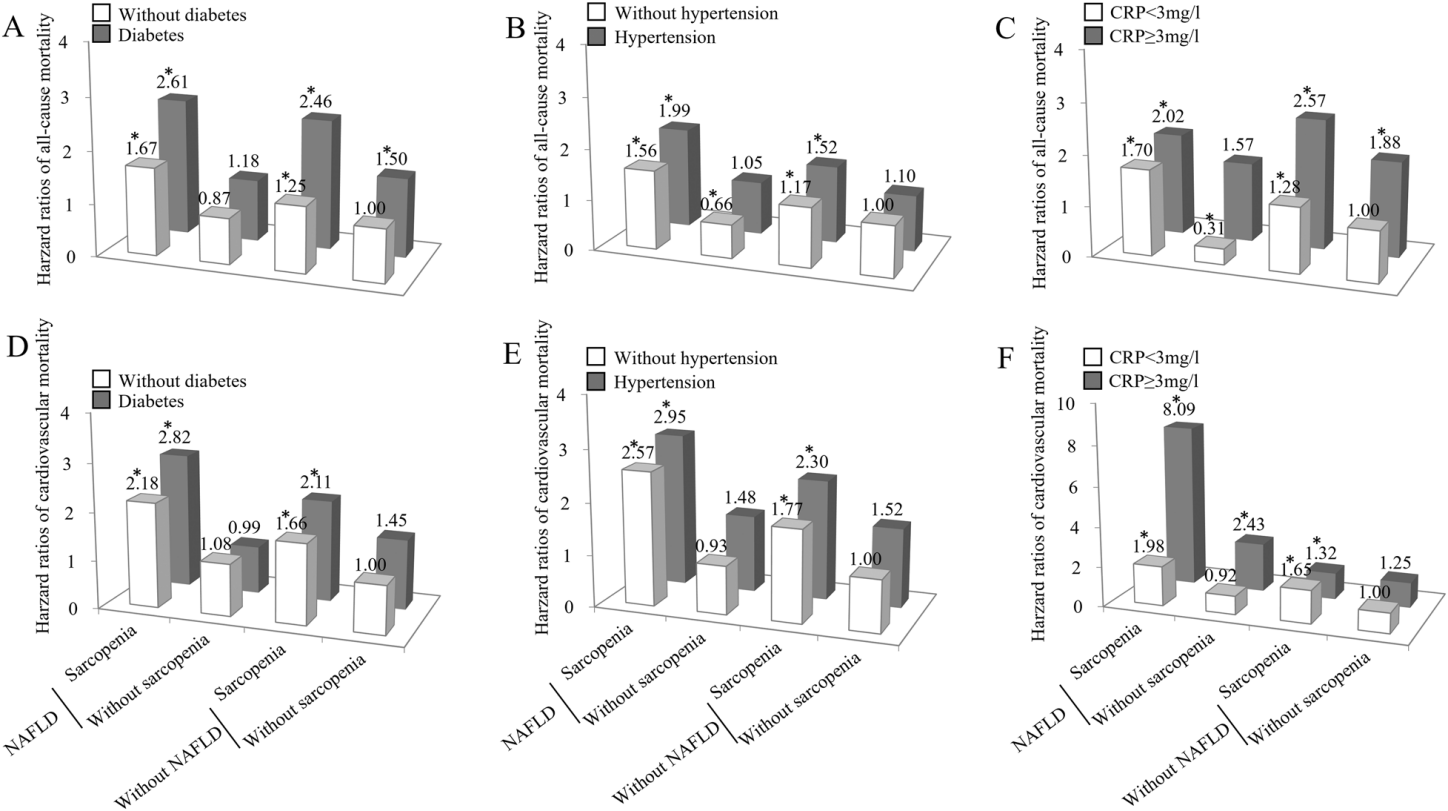
Joint effects of nonalcoholic fatty liver disease (NAFLD) and sarcopenia on all-cause (upper panel) and cardiovascular mortality (lower panel) after stratification for diabetes, hypertension, and inflammation status. *All-cause and cardiovascular mortality risks were significantly higher in sarcopenic subjects with NAFLD regardless of the status of diabetes, hypertension, and inflammation. Hazard ratios (95% confidence intervals) were adjusted for age, gender, race-ethnicity, education level, smoking and drinking status, body mass index, total cholesterol, triglyceride, HDL-cholesterol, and comorbid conditions such as stoke, coronary heart disease, and chronic obstructive pulmonary disease. Statistics and *P* values were showed in Supplementary Table S3.

Considering that diabetes, hypertension, and inflammation were well-established risk factors for all-cause and cardiovascular mortality, we explored the combined association of NAFLD and sarcopenia with all-cause and cardiovascular mortality after stratification by these conditions. NAFLD patients with sarcopenia conferred increased risks of all-cause and cardiovascular mortality, irrespective of the status of diabetes, hypertension, or inflammation (Fig. 3).

## Discussion

This nationally representative, older population-based study revealed the modulating effect of sarcopenia on the association of NAFLD with all-cause and cardiovascular mortality. Specifically, NAFLD was associated with increased risks of all-cause and cardiovascular mortality only among sarcopenic subjects. In contrast, NAFLD was a benign condition for all-cause and cardiovascular mortality among individuals without sarcopenia. This study further demonstrated that the magnitude of all-cause and cardiovascular mortality risk contributed by sarcopenia appeared to be much greater than the risk imparted by NAFLD. In this study, combined analyses broaden our understanding of risk factors’ relative influence on all-cause and cardiovascular mortality.

Investigations that disregarded the potential effect of sarcopenia on mortality concluded that NAFLD was associated with an increased mortality risk^4^. In contrast, other studies reported that mortality risk was not statistically different between subjects with and without NAFLD^5,28,29^. These conflicting data may be attributed, at least in part, to the inability to account for sarcopenia as a potential effect modifier. To date, investigations into sarcopenia as an effect modifier of the association of NAFLD with mortality are sparse^30^. Contrary to the benign prognosis of simple steatosis, NAFLD with advanced fibrosis correlates with increased all-cause and cardiovascular mortality^31,32^. A recent investigation provided strong evidence of a close relationship between sarcopenia, which was defined by decreased BMI-adjusted appendicular skeletal muscle mass, and liver fibrosis independently of obesity, insulin resistance, and liver enzyme levels among NAFLD subjects^33^. The method of adjustment of muscle mass determines the association of sarcopenia with functional and disability measures, insulin resistance, metabolic syndrome, and cardiovascular disease^34–36^. For example, skeletal mass adjusted for BMI or body weight was more associated with the presence of metabolic syndrome^37^, NAFLD predicting scores and advanced fibrosis^38^, while less cardiometabolic risk or an inverse correlation was noted when skeletal mass adjusted for height squared^34^. Our study used the height square-adjusted definition to define sarcopenia evidenced the negative relationship between SMI and the severity of hepatic fibrosis. Further studies are warranted to confirm the results. Sarcopenia may be a surrogate marker of fibrosis in the state of NAFLD. Hence, our findings suggest the necessity for an active assessment of sarcopenia status in NAFLD subjects.

Our finding that NAFLD is associated with increased mortality risk among sarcopenic subjects is consistent with the limited evidence^30^ and is of particular clinical importance. In the United States, NAFLD represents one of the most frequent causes of chronic liver disease and the most common indication for liver transplantation^39^. Worse is that sarcopenia assessed by computed tomography scan predicts negative preoperative and postoperative outcomes in liver transplant patients^40^. No approved pharmacotherapies for NAFLD are currently available. Considering that increased protein intake, vitamin D supplementation, as well as resistance training is a potent therapy that improves sarcopenia^41–43^, increasing the skeletal muscle mass through a high protein meal intake, vitamin D supplementation, and resistance exercise may be a promising potential treatment option for NAFLD. Awareness of the accompanying sarcopenia in NAFLD persons when deciding the optimal time for commencing therapeutic interventions is undoubtedly necessary to prevent disease progression and improve the negative long-term outcomes in NAFLD patients.

Information on the relative influence of NAFLD and sarcopenia on mortality risk is limited. We observed a significantly increased mortality risk associated with sarcopenia compared with no increase in the risk associated with NAFLD. Further, we noted that NAFLD was associated with increased risks of all-cause and cardiovascular mortality only in the presence of sarcopenia. Taken together, the magnitude of association with muscle mass is much greater than with NAFLD. It has been postulated that muscle mass can affect insulin metabolism by releasing healthy myokines such as irisin^44^. A preserved muscle mass may therefore be a key mechanism to increase the secretion of the favorable myokines and subsequently reduce the mortality risk. Further studies are needed to clarify the protective effects of sarcopenia management on the amelioration of NAFLD-associated mortality.

Our study further demonstrated that NAFLD combined with sarcopenia presented the greatest all-cause and cardiovascular mortality risk. However, the mortality risk associated with the combined NAFLD and sarcopenia did not exceed the sum of their individual risk, suggesting that NAFLD and sarcopenia have an interactive rather than additive effect on mortality. Although NAFLD and sarcopenia are viewed as 2 independent variables, they may be interacting with each other and contributing to the same causal pathway leading to all-cause and cardiovascular mortality.

This study has several strengths. A population-based analysis using well-examined nationwide data ensures the statistical reliability of our results and generalizability of the data. In addition, our study provides solid evidence of an independent association of combined NAFLD and sarcopenia with mortality after adjusting for a variety of important confounders including insulin resistance, inflammatory markers, and comorbid conditions.

We acknowledge several limitations. First, sarcopenia definition used in the present study is an incomplete definition as sarcopenia definition in the study did not cover the muscle strength aspect^45^. Although, there existed a population of adults who have declined physical function because they have low muscle mass, loss of muscle function, including usual gait speed, and mass are not reciprocally related. Impaired functionality itself is a mortality indicator^46^. Low muscle mass is one of many contributing factors in the development of functional limitations^46^. Further studies based on standard definition of sarcopenia that encompass muscle strength are warranted. Second, we classified skeletal muscle mass and gait speed into 2 levels to maximize statistical power. However, there is no apparent threshold in graded associations between these two measures and mortality. Third, since the sample is based on a cohort of participants aged ≥ 60 years, extrapolating results to younger adults should be done cautiously. Lastly, the observational study design makes it difficult to infer the causality or temporality between NAFLD/sarcopenia and risks of all-cause and cardiovascular mortality.

## Conclusion

NAFLD is associated with an increased all-cause and cardiovascular mortality risk in sarcopenic older adults. The association of NAFLD and mortality is less clear in adults without sarcopenia. These findings underscore the critical importance of sarcopenia as a determinant of mortality in NAFLD persons.

## Supplementary Information

Supplementary Informations.

## Abbreviations

NAFLD: Nonalcoholic fatty liver disease
BMI: Body mass index
WC: Waist circumference
BP: Blood pressure
TC: Total cholesterol
CRP: C-reactive protein
HOMA-IR: Homeostasis model assessment of insulin resistance
BIA: Bioelectrical impedance analysis
SMI: Skeletal muscle index
HR: Hazard ratio
FM: Fat mass
FFM: Fat-free mass
ICD: Injuries and causes of death
NHANES: The National Health and Nutrition Examination Surveys
NCHS: The National Center for Health Statistics
NDI: National Death Index
