# Supplementary material for: Sarcopenia modifies the associations of nonalcoholic fatty liver disease with all-cause and cardiovascular mortality among older adults
Source: Sci Rep. 2021 Aug 2;11:15647. doi: 10.1038/s41598-021-95108-1 (PMC8329219; doi:10.1038/s41598-021-95108-1)

**Sarcopenia modifies the associations of nonalcoholic fatty liver disease with** **all-cause and cardiovascular mortality among older adults**

Xingxing Sun^1^, Zhelong Liu^2, 3^, Fuqiong Chen^2, 3*^, Tingting Du^2, 3*^

^1^Department of Anesthesiology, Tongji Hospital, Tongji Medical College, Huazhong University of Science and Technology, Wuhan 430030, China

^2^Department of Endocrinology, Tongji Hospital, Tongji Medical College, Huazhong University of Science and Technology, Wuhan 430030, China.

^3^Branch of national clinical research center for metabolic diseases, Hubei.

^*^Corresponding author: Tingting Du, Department of Endocrinology, Tongji Hospital, Tongji Medical College, Huazhong University of Science and Technology, Wuhan, Hubei Province, 430030，PR China. E-mail: [aduttsxx@163.com](mailto:aduttsxx@163.com); Tel & Fax: 86 27 85491425. Fuqiong Chen, Department of Endocrinology, Tongji Hospital, Tongji Medical College, Huazhong University of Science and Technology, Wuhan 430030, China. Tel and Fax: +86 27 83661425, E-mail: [279251799@qq.com](mailto:279251799@qq.com)

Supplementary Table S1 Characteristics of the study participants with and without gait speed data.

|  | Without Gait speed | With Gait speed | P |
| --- | --- | --- | --- |
| N | 502 | 2446 |  |
| Age, years | 66.2±0.4 | 66.6±0.1 | 0.199 |
| Men, % | 51.7 | 42.4 | 0.032 |
| Non-Hispanic white | 89.7 | 83.8 | 0.116 |
| Non-Hispanic black | 4.5 | 8.0 | 0.051 |
| Mexican American | 1.4 | 2.6 | 0.040 |
| Body mass index, kg/m^2^ | 26.9±0.3 | 27.5±0.2 | 0.061 |
| Waist circumference, cm | 96.5±1.2 | 97.9±0.4 | 0.220 |
| Systolic blood pressure, mmHg | 136.5±1.3 | 136.1±0.4 | 0.745 |
| Diastolic blood pressure, mmHg | 75.4±0.8 | 74.9±0.3 | 0.601 |
| Plasma glucose, mmol/l | 5.8±0.2 | 5.9±0.1 | 0.813 |
| HbA1c, % | 5.6±0.1 | 5.8±0.0 | 0.042 |
| Total cholesterol, mmol/l | 6.0±0.1 | 5.9±0.0 | 0.206 |
| Triglycerides, mmol/l | 2.0±0.2 | 1.9±0.0 | 0.715 |
| HDL-cholesterol, mmol/l | 1.3±0.0 | 1.3±0.0 | 0.552 |
| LDL-cholesterol, mmol/l | 3.8±0.1 | 3.7±0.0 | 0.464 |
| C-reactive protein, mg/l | 1.0±0.1 | 1.0±0.1 | 0.829 |
| HOMA-IR | 2.9±0.4 | 3.9±0.2 | 0.021 |
| Skeletal muscle index, kg/m^2^ | 8.8±0.2 | 8.4±0.1 | 0.230 |

NAFLD, nonalcoholic fatty liver disease; HbA1c, hemoglobin A1c; HDL, high density lipoprotein; LDL, low density lipoprotein; HOMA-IR, homeostasis model assessment of insulin resistance

Supplementary Table S2 All-cause mortality during 16.8 years of follow-up according to combinations of nonalcoholic fatty liver disease (NAFLD) and sarcopenia status defined by fat mass to fat-free mass ratio

|  | Without NAFLD | | NAFLD | | | |  |
| --- | --- | --- | --- | --- | --- | --- | --- |
|  | Sarcopenia |  | Without sarcopenia |  | Sarcopenia |  | |
| All-cause mortality |  |  |  |  |  |  | |
| Model 1 | 1.18 (1.04-1.47) | *P*=0.035 | 1.00 (0.80-1.22) | *P*=0.953 | 1.43 (1.20-1.70) | *P*<.0001 | |
| Model 2 | 1.06 (1.01-1.26) | *P*=0.029 | 0.89 (0.73-1.09) | *P*=0.295 | 1.27 (1.02-1.56) | *P*=0.026 | |
| Model 3 | 1.07 (1.02-1.27) | *P*=0.018 | 0.89 (0.73-1.10) | *P*=0.303 | 1.28 (1.03-1.59) | *P*=0.026 | |
| Model 4 | 1.04 (1.00-1.26) | *P*=0.022 | 0.92 (0.68-1.26) | *P*=0.637 | 1.06 (1.01-1.45) | *P*=0.031 | |
| Model 5 | 1.03 (1.01-1.20) | *P*=0.030 | 0.80 (0.71-1.00) | *P*=0.186 | 1.31 (1.06-1.63) | *P*=0.012 | |
| Model 6 | 1.04 (1.01-1.28) | *P*=0.027 | 0.93 (0.75-1.10) | *P*=0.455 | 1.19 (1.03-1.48) | *P*=0.011 | |

Subjects without sarcopenia or NAFLD were served as the reference group.

Model 1 was adjusted for age, gender, race-ethnicity, and education level.

Model 2 was adjusted for smoking and drinking status, body mass index, hypertension, and diabetes in addition to the factors included in model 1.

Model 3 was adjusted for total cholesterol, triglyceride, and HDL-cholesterol in addition to the factors included in model 2.

Model 4 was adjusted for C-reactive protein in addition to the factors included in model 3.

Model 5 was adjusted for HOMA-IR in addition to the factors included in model 3.

Model 6 was adjusted for comorbid conditions such as stoke, coronary heart disease, and chronic obstructive pulmonary disease in addition to the factors included in model 3.

Supplementary Table S3 Joint effects of nonalcoholic fatty liver disease (NAFLD) and sarcopenia on all-cause and cardiovascular (CV) mortality in subgroup analysis

| Groups | | | All-cause mortality | | CV mortality |  |
| --- | --- | --- | --- | --- | --- | --- |
| Age 60~69 years (1652) | Without NAFLD | Without sarcopenia | 1 |  | 1 |  |
|  |  | With sarcopenia | 1.41 (1.03-2.02) | *P*=0.042 | 2.11 (1.10-4.05) | *P*=0.023 |
|  | With NAFLD | Without sarcopenia | 0.86 (0.69-1.08) | *P*=0.221 | 0.93 (0.58-1.40) | *P*=0.789 |
|  |  | With sarcopenia | 3.17 (2.25-4.40) | *P*<.0001 | 3.64 (2.09-6.30) | *P*<.0001 |
| Age ≧70 years (794) | Without NAFLD | Without sarcopenia | 2.31 (1.92-2.78) | *P* <.0001 | 2.31 (1.54-3.47) | *P*<.0001 |
|  |  | With sarcopenia | 2.85 (2.13-3.81) | *P* <.0001 | 2.87 (1.62-5.07) | *P*<.001 |
|  | With NAFLD | Without sarcopenia | 1.98 (1.50-2.62) | *P* <.0001 | 2.42 (1.47-3.99) | *P*=0.001 |
|  |  | With sarcopenia | 2.50 (1.68-3.72) | *P* <.0001 | 3.63 (1.81-7.27) | *P*<.001 |
| Non-Hispanic White (1246) | Without NAFLD | Without sarcopenia | 1 |  | 1 |  |
|  |  | With sarcopenia | 1.36 (1.05-1.78) | *P*=0.019 | 1.82 (1.10-3.02) | *P*=0.019 |
|  | With NAFLD | Without sarcopenia | 0.80 (0.70-1.08) | *P*=0.241 | 1.06 (0.72-1.57) | *P*=0.756 |
|  |  | With sarcopenia | 1.66 (1.17-2.36) | *P*=0.004 | 2.35 (1.37-4.02) | *P*=0.002 |
| Non-Hispanic Black, Mexican-American, and other (1200) | Without NAFLD | Without sarcopenia | 1.02 (0.70-1.49) | *P*=0.888 | 1.18 (0.49-2.84) | *P*=0.696 |
|  |  | With sarcopenia | 1.11 (1.02-1.66) | *P*=0.024 | 1.16 (1.08-2.61) | *P*=0.033 |
|  | With NAFLD | Without sarcopenia | 0.61 (0.34-1.08) | *P*=0.091 | 0.57 (0.20-1.40) | *P*=0.237 |
|  |  | With sarcopenia | 1.91 (1.07-3.41) | *P*=0.028 | 2.19 (1.47-4.10) | *P*=0.026 |
| Without diabetes (1927) |  | Without sarcopenia | 1 | / | 1 |  |
|  | Without NAFLD | With sarcopenia | 1.25 (1.07-1.60) | *P*=0.021 | 1.66 (1.01-2.74) | *P*=0.044 |
|  | With NAFLD | Without sarcopenia | 0.87 (0.70-1.07) | *P*=0.213 | 1.08 (0.72-1.61) | *P*=0.699 |
|  |  | With sarcopenia | 1.67 (1.14-2.46) | *P*=0.008 | 2.18 (1.20-3.98) | *P*=0.010 |
| Diabetes (519) | Without NAFLD | Without sarcopenia | 1.50 (1.15-1.96) | *P*=0.003 | 1.45 (0.84-2.53) | *P*=0.179 |
|  |  | With sarcopenia | 2.46 (1.72-3.51) | *P*<.0001 | 2.11 (1.18-4.86) | *P*=0.027 |
|  | With NAFLD | Without sarcopenia | 1.18 (0.89-1.57) | *P*=0.244 | 0.99 (0.53-1.00) | *P*=0.978 |
|  |  | With sarcopenia | 2.61 (1.59-4.29) | *P*=.0001 | 2.82 (1.34-5.91) | *P*=0.006 |
| Without hypertension (942) | Without NAFLD | Without sarcopenia | 1 |  | 1 |  |
|  |  | With sarcopenia | 1.17 (1.02-1.75) | *P*=0.034 | 1.77 (1.13-4.24) | *P*=0.022 |
|  | With NAFLD | Without sarcopenia | 0.66 (0.47-0.94) | *P*=0.022 | 0.93 (0.48-1.79) | *P*=0.834 |
|  |  | With sarcopenia | 1.56 (1.09-2.64) | *P*=0.039 | 2.57 (1.21-5.40) | *P*=0.014 |
| Hypertension (1504) | Without NAFLD | Without sarcopenia | 1.10 (0.89-1.37) | *P*=0.340 | 1.52 (0.97-2.39) | *P*=0.065 |
|  |  | With sarcopenia | 1.52 (1.12-2.00) | *P*=0.006 | 2.30 (1.29-4.38) | *P*=0.005 |
|  | With NAFLD | Without sarcopenia | 1.05 (0.82-1.36) | *P*=0.666 | 1.48 (0.88-2.49) | *P*=0.135 |
|  |  | With sarcopenia | 1.99 (1.34-2.95) | *P*=0.001 | 2.95 (1.40-5.84) | *P*=0.002 |
| CRP < 3 (2389) | Without NAFLD | Without sarcopenia | 1 |  | 1 |  |
|  |  | With sarcopenia | 1.28 (1.01-1.61) | *P*=0.038 | 1.65 (1.04-2.61) | *P*=0.031 |
|  | With NAFLD | Without sarcopenia | 0.31 (0.11-0.53) | *P*=0.023 | 0.92 (0.64-1.33) | *P*=0.695 |
|  |  | With sarcopenia | 1.70 (1.22-2.36) | *P*=0.002 | 1.98 (1.18-3.30) | *P*=0.009 |
| CRP ≥ 3 (57) | Without NAFLD | Without sarcopenia | 1.88 (1.20-2.90) | *P*=0.006 | 1.25 (0.48-3.23) | *P*=0.641 |
|  |  | With sarcopenia | 2.57 (1.17-5.65) | *P*=0.018 | 1.32 (1.11-5.52) | *P*=0.032 |
|  | With NAFLD | Without sarcopenia | 1.57 (0.81-3.03) | *P*=0.174 | 2.43 (1.31-4.50) | *P*=0.005 |
|  |  | With sarcopenia | 2.02 (1.24-4.80) | *P*=0.025 | 8.09 (3.88-16.8) | *P*<.0001 |

Data were shown as Hazard ratios (95% confidence intervals). Hazard ratios (95% confidence intervals) of all-cause and cardiovascular mortality were adjusted for age, gender, education level, smoking and drinking status, body mass index, total cholesterol, triglyceride, HDL-cholesterol, and comorbid conditions such as stoke, coronary heart disease, and chronic obstructive pulmonary disease.

Because of the infrequency of participants with Non-Hispanic Black (537), Mexican-American race-ethnicity (585), and other race-ethnicity (78), we collapsed Non-Hispanic Black, Mexican-American, and other into a single race-ethnicity.

Supplementary Figure S1

Skeletal mass index across quartiles of Fibrosis‐4 score


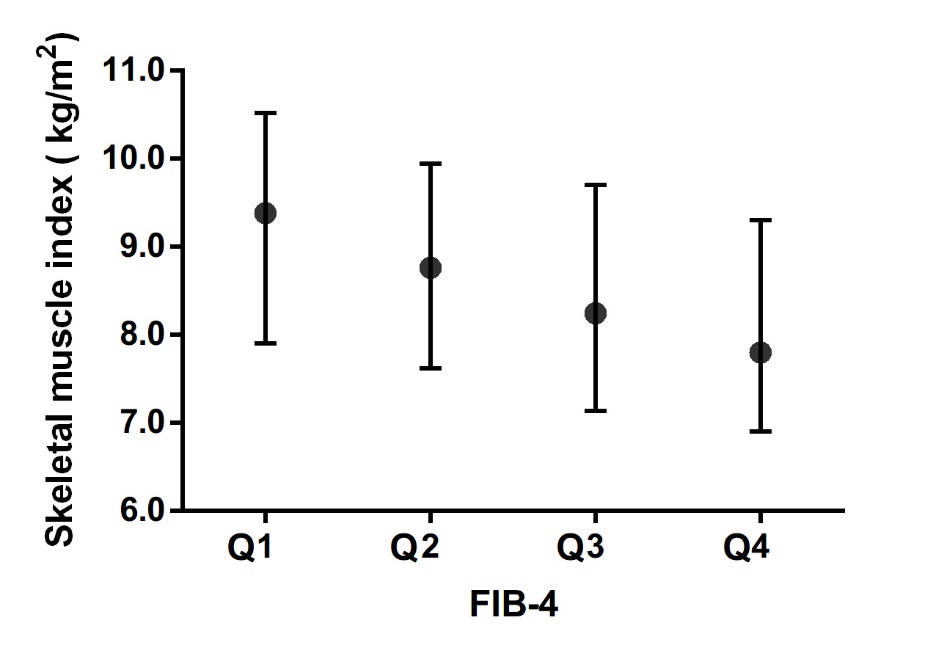

Supplement: Supplementary file 1 — Supplementary Informations. [file 41598_2021_95108_MOESM1_ESM.docx]
